# Epidemiology of the diabetes-cardio-renal spectrum: a cross-sectional report of 1.4 million adults

**DOI:** 10.1186/s12933-022-01521-9

**Published:** 2022-06-10

**Authors:** Meir Schechter, Cheli Melzer Cohen, Ilan Yanuv, Aliza Rozenberg, Gabriel Chodick, Johan Bodegård, Lawrence A. Leiter, Subodh Verma, Hiddo J. Lambers Heerspink, Avraham Karasik, Ofri Mosenzon

**Affiliations:** 1 Diabetes Unit, Department of Endocrinology and Metabolism, Hadassah Medical Center, P.O. Box 12000, 9112001 Jerusalem, Israel; 2 Faculty of Medicine, Hebrew University of Jerusalem, Jerusalem, Israel; 3 Department of Clinical Pharmacy and Pharmacology, University Medical Center Groningen, University of Groningen, Groningen, The Netherlands; 4 Maccabi Institute for Research and Innovation, Maccabi Healthcare Services, Tel-Aviv, Israel; 5 School of Public Health Sackler, Faculty of Medicine, Tel Aviv University, Tel Aviv, Israel; 6 Cardiovascular, Renal and Metabolism, Medical Department, BioPharmaceuticals, AstraZeneca, Oslo, Norway; 7 Li Ka Shing Knowledge Institute, St. Michael’s Hospital, University of Toronto, Toronto, ON Canada; 8 Division of Cardiac Surgery, St. Michael’s Hospital, University of Toronto, Toronto, ON Canada; 9 Tel Aviv University, Tel Aviv, Israel

**Keywords:** Type-2 Diabetes, Heart Failure, Chronic Kidney Disease, Cross-sectional Study, Epidemiology, Diabetes-cardio-renal Spectrum

## Abstract

**Background:**

Type-2 diabetes (T2D), chronic kidney disease, and heart failure (HF) share epidemiological and pathophysiological features. Although their prevalence was described, there is limited contemporary, high-resolution, epidemiological data regarding the overlap among them. We aimed to describe the epidemiological intersections between T2D, HF, and kidney dysfunction in an entire database, overall and by age and sex.

**Methods:**

This is a cross-sectional analysis of adults ≥ 25 years, registered in 2019 at Maccabi Healthcare Services, a large healthcare maintenance organization in Israel. Collected data included sex, age, presence of T2D or HF, and last estimated glomerular filtration rate (eGFR) in the past two years. Subjects with T2D, HF, or eGFR < 60 mL/min/1.73 m^2^ were defined as within the diabetes-cardio-renal (DCR) spectrum.

**Results:**

Overall, 1,389,604 subjects (52.2% females) were included; 445,477 (32.1%) were 25– < 40 years, 468,273 (33.7%) were 40– < 55 years, and 475,854 (34.2%) were ≥ 55 years old. eGFR measurements were available in 74.7% of the participants and in over 97% of those with T2D or HF. eGFR availability increased in older age groups. There were 140,636 (10.1%) patients with T2D, 54,187 (3.9%) with eGFR < 60 mL/min/1.73m^2^, and 11,605 (0.84%) with HF. Overall, 12.6% had at least one condition within the DCR spectrum, 2.0% had at least two, and 0.23% had all three. Cardiorenal syndrome (both HF and eGFR < 60 mL/min/1.73m^2^) was prevalent in 0.40% of the entire population and in 2.3% of those with T2D. In patients with both HF and T2D, 55.2% had eGFR < 60 mL/min/1.73m^2^ and 15.8% had eGFR < 30 mL/min/1.73m^2^. Amongst those within the DCR spectrum, T2D was prominent in younger participants, but was gradually replaced by HF and eGFR < 60 mL/min/1.73m^2^ with increasing age. The congruence between all three conditions increased with age.

**Conclusions:**

This large, broad-based study provides a contemporary, high-resolution prevalence of the DCR spectrum and its components. The results highlight differences in dominance and degree of congruence between T2D, HF, and kidney dysfunction across ages.

**Supplementary Information:**

The online version contains supplementary material available at 10.1186/s12933-022-01521-9.

## Introduction

Globally, approximately 537 million subjects have type-2 diabetes [1], 697 million have chronic kidney disease (CKD)[2], and 64 million have heart failure (HF) [3]. These conditions form together vicious pathophysiological circles, and the presence of one may contribute to the development of the others [4, 5]. For example, type-2 diabetes contributes to the development of both HF and CKD [6], and HF can lead to a decline of kidney functions and vice-versa as part of the cardiorenal syndrome [4, 7, 8].

For several years, treatment options for type-2 diabetes, CKD, and HF did not necessarily overlap. For example, thiazolidinediones benefit glycemic control in patients with type-2 diabetes; and endothelin receptor antagonists improve kidney outcomes in patients with CKD. Yet, both were associated with increased risk for HF [9, 10]. Renin–angiotensin–aldosterone system blockade improves outcomes in patients with HF or CKD [11–13] but does not affect glycemic control. More recently, sodium–glucose co-transporter 2 inhibitors (SGLT2i) were shown to improve glycemic control and reduce the onset of HF and CKD in patients with type-2 diabetes, as well as improve outcomes in patients with CKD or HF with or without type-2 diabetes [14–21]. Thus, SGLT2i therapy benefits patients across the “diabetes-cardio-renal spectrum” (DCR spectrum) [22]. Although the epidemiological characteristics of these conditions have been described [1–3], there is a paucity of contemporary high-quality epidemiological data regarding the overlap among the different components of the DCR spectrum across sex and ages subgroups. This evidence gap translates into a clinical obstacle when assessing a specific patient’s probability of having one or more DCR spectrum components.

We performed a cross-sectional analysis of adults registered at Maccabi Healthcare Services (MHS) in Israel. The database enjoys very high yearly retention (99%), validated type-2 diabetes and HF registries [23, 24], and high granularity of estimated glomerular filtration rate (eGFR) measurements. We used these advantages to describe with high resolution the contemporary (2019) prevalence and overlap of different components of the DCR spectrum by age and sex categories.

## Methods

### Study design and populations

This is a cross-sectional and contemporary analysis of the MHS database, Israel’s second-largest healthcare maintenance organization (HMO). It was designed as a broad-based analysis of an entire database to limit population selection biases (Additional file 1: Fig. S1). Adults 25 years or older, who were members of MHS between January 1, 2019, and December 31, 2019, were included. This age was selected since in Israel, most young men and women are required to serve in the army for several years, a period in which they are not members of the general HMOs system. Excluded are patients who were part of a type-1 diabetes registry, identified as < 26 years upon entry to the diabetes registry, and having > 4 insulin purchases per year upon diabetes diagnosis without an oral glucose-lowering agent. This age cutoff was selected since type-1 diabetes incidence reduces by 60–80% in those older than 26, compared to those in their teen years [25]. While this definition may lead to the inclusion of patients who were diagnosed with type-1 diabetes at older ages, their numbers are expected to be diluted among the significantly larger incidence of type-2 diabetes.

The study received IRB approval from MHS Institutional Review Broad committee at MHS. Due to de-identified data extraction, informed consent was not requested by the IRB. Financial support for data accrual was obtained from AstraZeneca as part of the CAREME initiative. However, the conceptualization of this analysis and its execution was performed by the primary investigators. No payment was received to analyze the data or to write this manuscript.

### Variable definition

Data was extracted from January 1, 2018, until December 31, 2019. Collected data per each participant included sex, age, last eGFR measurement (between January 1, 2018, to December 31, 2019), and inclusion in type-2 diabetes or HF registry before December 31, 2019 [23, 24].

The purpose of the MHS registries is to benefit disease management at single-physician levels and in the entire HMO scale. Inclusion in the diabetes registry was based on a validated algorithm [24, 26]. This algorithm combines information of laboratory measurements (HbA1c and fasting plasma glucose), dispensed medication (insulin or oral glucose-lowering agents), and diagnoses made by expert physicians (see more elaboration in the Additional file 1:Methods). Inclusion in HF registry was defined as HF diagnoses by a hospital or a community cardiologist on two occasions or more. For this purpose, the following International Classification of Diseases, 9th revision (ICD-9) codes were used: 404.x, 428.x, 398.91 and 785.51. This definition was chosen after a validation process generated with creating the cardiovascular disease registry [23]. Physicians may request to exclude patients from the registries if they were captured with the algorithm by mistake.

Creatinine was measured in MHS central laboratories by compensated Jaffe methodology. Only measurements in out-patient settings were used to reduce biases associated with acute in-patient admissions. eGFR was calculated by the CKD-EPI Equation [27]. The diabetes-cardio-renal (DCR) spectrum was defined as either type-2 diabetes, HF, and/or eGFR < 60 mL/min/1.73 m^2^ (CKD stage 3 or worse). The cardiorenal syndrome was defined as having both HF and eGFR < 60 mL/min/1.73 m^2^.

Age categories were used as follows: 25- < 40; 40- < 55; 55- < 65; 65- < 75; 75- < 85 and 85 + years. When required, the 25- < 40 and 40- < 55 years group were merged. The following eGFR categories were used > 90; 60- < 90; 30- < 60 or < 30 mL/min/1.73 m^2^, in accordance with the KDIGO classification.

### Statistical analysis

This is a descriptive analysis, and no formal hypotheses were tested. Presented are numbers or percentages of patients, as specifically indicated. We did not perform comparative tests among groups. Distribution within eGFR groups is calculated out of the total relevant population as stated, including those without measurement, to reduce biases associated with lower eGFR availability in healthier populations. Where expressly indicated, the eGFR distribution is calculated only of those with available eGFR values.

## Results

### Prevalence of type-2 diabetes, heart failure, and eGFR < 60 mL/min/1.73 m^2^ by age and sex

**Fig. 1:**
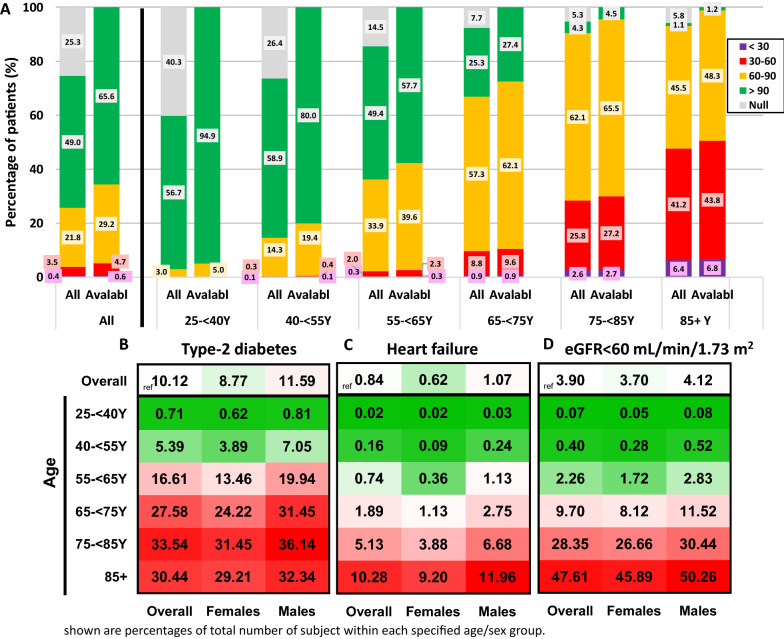
Prevalence of type-2 diabetes, heart failure, or reduced kidney functions (eGFR < 60 mL/min/1.73 m^2^) by age and sex. **A** Distribution of eGFR values by age groups in the MHS population, presented as percentage of the entire cohort (all) and of those with an available eGFR value during the past two years (‘avlable’). eGFR values are presented as mL/min/1.73 m^2^. **B** Prevalence of T2D; **C** HF; or **D** eGFR < 60 mL/min/1.73 m^2^ overall and by age and sex subgroups. Numbers indicate the percentage of patients out of the entire sex-/age-matched population in the MHS database. For each condition, greener indicates lower prevalence, redder indicates higher prevalence. White indicates the overall prevalence in the study population. *T2D* Type-2 diabetes, *HF* heart failure, *eGFR* estimated glomerular filtration rate, *MHS* Maccabi Healthcare Services

There were overall 1,389,604 subjects eligible to participate in this study; 52.2% were females. About a third (32.1%) aged 25- < 40 years, another third (33.7%) were 40- < 55 years old, and the rest were 55 years or older (Table 1). Most (74.7%) had an available eGFR measurement, and the eGFR availability increased in older age groups (Fig. 1A). Overall, there were 140,636 (10.12%) patients with type-2 diabetes, 54,187 (3.90%) patients with eGFR measurement below 60 mL/min/1.73 m^2^, and 11,605 (0.84%) patients with HF. Prevalence of all three conditions was higher in older age groups; while type-2 diabetes prevalence trajectory mainly increased at 40- < 75 years and plateaued in older age groups, HF and eGFR < 60 mL/min/1.73 m^2^ prevalence was markedly higher in those older than 65 years (Fig. 1B–D). More than half of patients with HF or with eGFR < 60 mL/min/1.73 m^2^ were > 75 years old, compared with less than a quarter of those with type-2 diabetes (Additional file 1: Fig. S2). Males had a numerically higher prevalence of all three conditions than females, overall and across all tested age groups (Fig. 1B–D).

**Table 1:** Sex and age distribution of patients registered at the MHS and included in this analysis

Age Group	Males	Females	Overall
25– < 40 years	217,537 (15.7)	227,940 (16.4)	445,477 (32.1)
40– < 55 years	223,198 (16.1)	245,075 (17.6)	468,273 (33.7)
55– < 65 years	106,082 (7.6)	112,010 (8.1)	218,092 (15.7)
65– < 75 years	76,081 (5.5)	87,449 (6.3)	163,530 (11.8)
75– < 85 years	31,527 (2.3)	39,174 (2.8)	70,701 (5.1)
85 + years	9,250 (0.7)	14,281 (1.0)	23,531 (1.7)
Overall	663,675 (47.8)	725,929 (52.2)	1,389,604 (100)

Presented as n (%)—number of patients (percentage of total). Numbers may not add up to 100% due to rounding

*MHS* Maccabi Healthcare Services

### Distributions and congruence of the diabetes-cardio-renal spectrum’s components

**Fig. 2:**
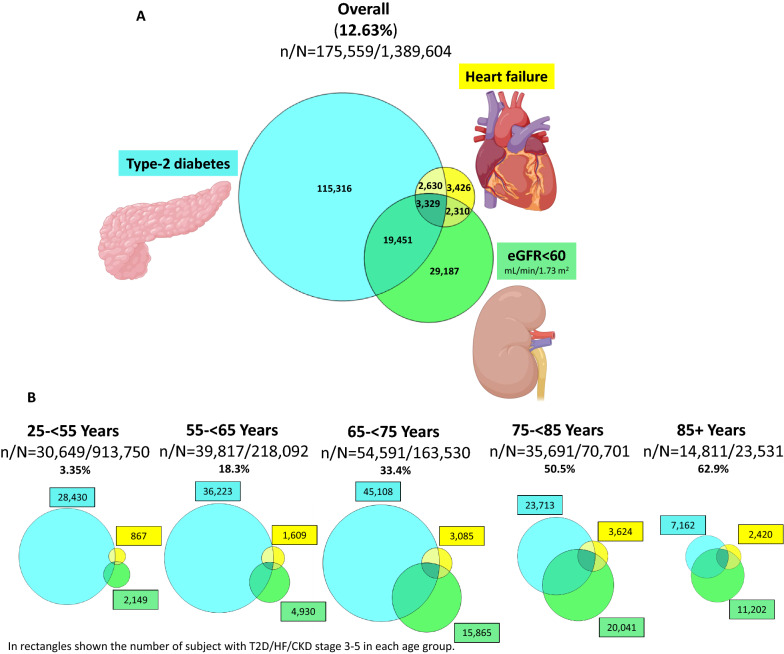
Overlap between components of the diabetes-cardio-renal (DCR) spectrum by age groups. Scaled Venn diagrams presenting the distribution of patients with T2D (blue), HF (yellow), and eGFR < 60 mL/min/1.73 m^2^ (green) and their degree of congruence **A** in the overall population and **B** by age groups. The area in each age group is proportionally scaled (per number of patients within the DCR spectrum) to the overall population, e.g., sum of “yellow/HF” area in all age groups approximately equals to the “yellow/HF” area in the general population. Created with BioRender.com with permission. *T2D* Type-2 diabetes, *HF* heart failure, *DCR* diabetes-cardio-renal, *eGFR* estimated glomerular filtration rate

**Fig. 3:**
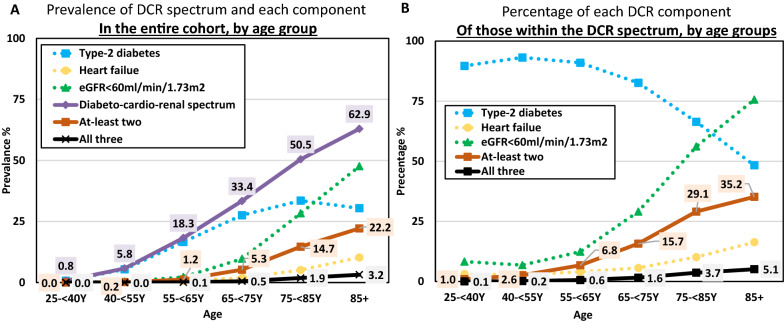
Prevalence of the diabetes-cardio-renal (DCR) spectrum and its components by age groups. **A** Prevalence of patients with at least one component of the DCR spectrum (defined as either T2D, HF, or eGFR < 60 mL/min/1.73 m^2^; purple), patients having at-least two (orange) or all three (black) components by different age groups—of the entire cohort’s subjects. **B** Prevalence of different components of the DCR spectrum (presented as in **A**), specifically in those that are within the DCR spectrum (i.e., have either T2D, HF, and/or eGFR < 60 mL/min/1.73 m^2^), by age groups. *T2D* Type-2 diabetes, *HF* heart failure, *DCR* diabetes-cardio-renal, *eGFR* estimated glomerular filtration rate

Overall, 12.63% (n = 175,559) had at least one component of the DCR spectrum (defined as either type-2 diabetes, HF and/or eGFR < 60 mL/min/1.73 m^2^), 1.99% (n = 27,630) had at least two conditions, and 0.23% (n = 3329) had all three (Fig. 2A). While the relative prevalence of the DCR spectrum gradually increased with age, reaching 62.9% in those 85 years or older (Figs. 2B, 3A), the numerically highest burden within this MHS cohort was in the 65- < 75 years subgroup (n = 54,591; 31.1% of the total number of patients within the DCR spectrum) (Fig. 2B). Generally, between-conditions congruence increased with older age (Figs. 2B, 3A, B). Of those within the DCR spectrum, the relative portion of patients with type-2 diabetes gradually reduced with older age, while the relative portion of HF or eGFR < 60 mL/min/1.73 m^2^ increased with age (Fig. 3B).

We looked at the prevalence of type-2 diabetes in patients with HF or eGFR < 60 mL/min/1.73 m^2^ overall and by different age groups. In younger subgroups, majority of patients with HF or eGFR < 60 mL/min/1.73 m^2^ did not have type-2 diabetes. The prevalence of type-2 diabetes in those populations increased with age and peaked in the 65- < 75 years subgroup. In participants older than 85 years, there was a numerically lower prevalence of concomitant type-2 diabetes in those with HF or eGFR < 60 mL/min/1.73 m^2^ (Additional file 1: Fig. S3).

### Prevalence of heart failure by the presence of type-2 diabetes and kidney functions

**Fig. 4:**
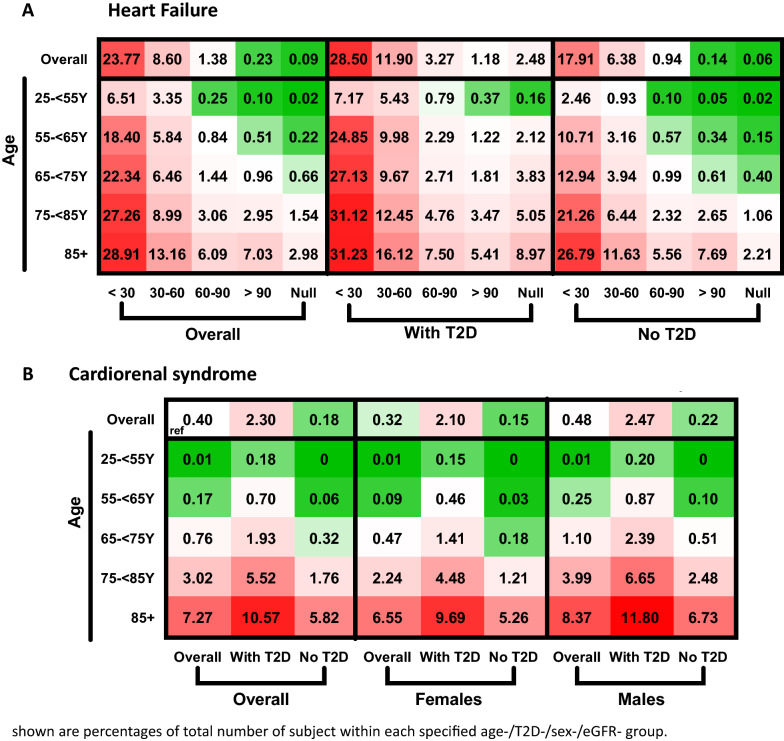
Prevalence of heart failure by presence of type-2 diabetes and kidney functions. **A** Prevalence of heart failure by age, presence of T2D, and eGFR category. **B** Prevalence of cardiorenal syndrome (defined as both heart failure and eGFR < 60 ml/min/1.73 m^2^) by age, sex, and presence of T2D. Numbers indicate the percentage of patients out of the entire sex-/age-/T2D-/eGFR category-matched population in the MHS. Greener indicates lower prevalence, and redder indicates higher prevalence. White (reference) represents the overall prevalence in the study population (0.84% for heart failure and 0.40% for cardiorenal syndrome). eGFR categories are in mL/min/1.73 m^2^. *T2D* Type-2 diabetes, *eGFR* estimated glomerular filtration rate, *MHS* Maccabi Healthcare Services

The calculated prevalence of HF increased in lower eGFR categories (0.23, 1.38, 8.60, or 23.77% in those with eGFR > 90, 60–90, 30–60 or > 30 mL/min/1.73 m^2^, respectively; Fig. 4A). These findings were generally similar across age, sex, and presence of type-2 diabetes groups (Fig. 4A, Additional file 1: Fig. S4). Overall, 5549 (0.40%) subjects had cardiorenal syndrome (both HF and eGFR < 60 mL/min/1.73 m^2^) and 2.30% of those with type-2 diabetes. The prevalence of cardiorenal syndrome increased with age and male sex, reaching 7.27% of participants older than 85 years (Fig. 4B).

Kidney functions in patients with type-2 diabetes, heart failure, both, or neither.

We plotted the distribution of eGFR categories in subjects with type-2 diabetes, HF, both, or neither. Over 97% of patients with HF or type-2 diabetes had an available eGFR measurement, and 72% of those with neither. In those without HF nor type-2 diabetes 0.17% had eGFR < 30 mL/min/1.73 m^2^, compared with 2.3% of those with type-2 diabetes, and 12.0% of those with HF (Additional file 1: Fig. S5A). Among patients with both HF and type-2 diabetes, 15.8% had eGFR < 30 mL/min/1.73 m^2^ relative to 8.2% of those with HF without type-2 diabetes (Additional file 1: Fig. S6A). Similar trends were observed across age groups (Additional file 1: Fig. S6B, S5B).

## Discussion

This contemporary 2019 cross-sectional descriptive study presents the association between type-2 diabetes, HF, and kidney function in 1.4 million Israeli adults. Amongst the entire cohort, the prevalence of type-2 diabetes, eGFR < 60 mL/min/1.73 m^2^ (CKD stage 3 or worse), and HF were 10.12%, 3.90%, and 0.84%, respectively. Overall, 12.63% had at least one of these conditions (defined as the diabetes-cardio-renal [DCR] spectrum); 2.22% had at least two conditions, and 0.23% had all three; 0.40% had the cardiorenal syndrome. Of those within the DCR spectrum, the relative portion of type-2 diabetes gradually reduced with older age, accompanied by a relative increase in HF and CKD stage 3–5. The degree of congruence between conditions increased with age. Overall, the analysis provides a comprehensive broad-based description of the epidemiological intersections between components of the DCR spectrum across age and sex groups.

The prevalence of diagnosed diabetes, HF, or CKD is highly variable among countries and regions. It depends on population age, lifestyle and culture, genetic variations, comorbidities, documentation quality, survival differences, and availability of screening programs [1–3, 26, 28]. To the best of our knowledge, this is the most updated and comprehensive documentation of HF prevalence in Israel [3, 28, 29]. The findings of this analysis suggest that HF prevalence in the study population (0.84%) is within the lower range compared to cohorts from other countries, usually ranging between 1–2% [3, 28, 29]. In accordance with previous reports, the observed total prevalence of type-2 diabetes in this Israeli population (10.1%) is similar to the global average (10.5%), higher than in Europe (9.2%), yet lower than in the USA (13.6%)[1, 26]. The prevalence of CKD in Israel, however, is relatively lower than in the rest of the world and specifically other high-middle socio-demographic index quintile countries [2]. Our findings of fairly granular eGFR measurements, especially in at-risk populations (those with older age, type-2 diabetes, and/or HF), suggest that underdiagnosis due to insufficient testing does not seem to explain these findings [26]. Overall, besides the point-prevalence of type-2 diabetes, HF, and kidney function, this analysis provides valuable data regarding the distribution of these conditions by age and sex with implications to policymaking.

Type-2 diabetes, CKD, and HF are components of one spectrum, which we collectively termed diabetes-cardio-renal (DCR) spectrum [7, 8, 22]. All three conditions share epidemiological and pathophysiological features [4, 5, 7, 8, 22]. The current analysis highlights type-2 diabetes as the main DCR component in younger participants, while HF and CKD become increasingly prevalent and more dominant among older adults. The results further show increased congruence between components of the DCR spectrum in older age groups. Previous cohort studies demonstrated that having one DCR component is associated with the development of the others [4, 6, 30–33], and that presence of more than one component is associated with a higher risk for adverse clinical outcomes and mortality [6, 30, 34, 35]. Accordingly, the prevalence of cardiorenal syndrome, which constitutes a patient group at especially high risk, increased steeply in those older than 75 years, especially in those with type-2 diabetes [6]. All in all, this high-resolution description may assist in evaluating individual patients’ risk of having one or more components of the DCR spectrum.

SGLT2i were shown to improve the prognosis of all three conditions across disease stages and phenotypes [14–20]. Early findings suggest that SGLT2i can also prevent the development of new HF, CKD, and type-2 diabetes in patients already having one component of the DCR spectrum [20, 21, 36], indicating that SGLT2i may interfere with the vicious cycles illustrated by Braunwald [4]. This analysis provides valuable estimations of the number of patients on the DCR spectrum who may benefit from treatment with SGLT2i, although limited by lack of albuminuria data.

For many years, resources were invested in developing new drugs that target single components of the DCR spectrum, resulting in a wide array of medications that improve glycemia, reduce HF-associated risk, or have some degree of kidney protection. More recently, however, increasing efforts have tried to find drugs and especially combination therapies that may benefit specific groups of patients within the DCR spectrum [37–39]. Further pursuing this line of research may be faster, more cost-effective, and result in better use of the currently approved therapies. Our findings support these attempts by providing a general epidemiological framework of patients populations that could benefit from such efforts.

## Study limitations

This study includes data from one HMO; therefore, the external validity of the findings to other populations, especially non-Caucasians, is limited. However, Israel’s population has relatively high genetic variability due to large communities of immigrants and their descendants. The analysis is solely descriptive due to its cross-sectional nature; thus, it does not allow to conclude regarding causality. The retrospective nature of this analysis, and its reliance on registries, may result in diagnoses misidentification in some cases. Many relevant variables such as body mass index, HbA1c, albuminuria status, ejection fraction, HF etiology or severity, prediabetes, diabetes retinopathy or other comorbidities, and medications were not included. Accordingly, the definition of cardiorenal syndrome relies on the coexistence of HF ICD-9 diagnosis and eGFR < 60 mL/min/1.73 m^2^, without considering other markers of heart or kidney disease. While some eGFR measurements are lacking, this group includes mostly younger and healthier populations. To address this issue, we presented data both including as well as disregarding the missing eGFR values.

## Conclusion

This huge, broad-based study of 1.4 million adults provides a contemporary 2019, high-resolution prevalence of the different components of the diabetes-cardio-renal spectrum. The findings highlight differences in the degree of congruence among type-2 diabetes, HF, and kidney dysfunction across age and sex groups. The results may assist in estimating patients’ and populations’ probability of having one or more components of the DCR spectrum, with practical implications for policymaking, clinical research, and daily patient care.

## Supplementary Information

**Additional file 1: Figure S1.** CONSORT diagram of subjects included in this analysis. **Figure S2.** Distribution by age of T2D, heart failure, and eGFR<60 mL/min/1.73 m^2^. **Figure S3.** Prevalence of T2D in patients with heart failure or eGFR<60 mL/min/1.73 m^2^ overall and at different age groups. **Figure S4.** Prevalence of heart failure by age, presence of T2D and kidney functions—in female and male participants separately. **Figure S5.** Distribution of patients within different kidney functions categories by presence of T2D, heart failure, or neither. **Figure S6.** Distribution of kidney functions in patients with heart failure by the presence of T2D.

## Data Availability

The datasets used and/or analysed during the current study are available from the corresponding author on reasonable request.

## Abbreviations

CKD: Chronic kidney disease
DCR: Diabetes-cardio-renal
eGFR: Estimated glomerular filtration rate
HF: Heart failure
HMO: Healthcare maintenance organization
ICD-9: International Classification of Diseases, 9th revision
KDIGO: Kidney Disease Improving Global Outcomes
MHS: Maccabi Healthcare Services
SGLT2i: Sodium/glucose cotransporter 2 inhibitors
T2D: Type-2 diabetes
