# Supplementary material for: Epidemiology of the diabetes-cardio-renal spectrum: a cross-sectional report of 1.4 million adults
Source: Cardiovasc Diabetol. 2022 Jun 10;21:104. doi: 10.1186/s12933-022-01521-9 (PMC9188046; doi:10.1186/s12933-022-01521-9)

**Epidemiology of the Diabetes Cardio-Renal Spectrum: A Cross-Sectional Report of 1.4 Million Adults**

Meir Schechter ^1,2,3^ MD PhD, Cheli Melzer-Cohen ^4^ MSc, Ilan Yanuv ^1,2^ MSc, Aliza Rozenberg ^1,2^ MA, Gabriel Chodick ^4,5^ PhD MHA, Johan Bodegård ^6^ MD PhD, Lawrence A. Leiter ^7^ MD, Subodh Verma ^8^ MD PhD, Hiddo J. L. Heerspink ^3^ PhD PharmD, Avraham Karasik ^4,9^ MD, Ofri Mosenzon^*^ ^1,2^ MD MSc

^1^ Diabetes Unit, Department of Endocrinology and Metabolism, Hadassah Medical Center, Jerusalem, Israel

^2^ Faculty of Medicine, Hebrew University of Jerusalem, Jerusalem, Israel

^3^ Department of Clinical Pharmacy and Pharmacology, University Medical Center Groningen, University of Groningen, Groningen, The Netherlands

^4^ Maccabi Institute for Research and Innovation, Maccabi Healthcare Services, Tel-Aviv, Israel

^5^ School of Public Health Sackler, Faculty of Medicine, Tel Aviv University, Tel Aviv, Israel

^6^ Cardiovascular, Renal and Metabolism, Medical Department, BioPharmaceuticals, AstraZeneca, Oslo, Norway

^7^ Li Ka Shing Knowledge Institute, St. Michael’s Hospital, University of Toronto, Toronto, ON, Canada

^8^ Division of Cardiac Surgery, St. Michael’s Hospital, University of Toronto, Toronto, ON, Canada

^9^ Tel Aviv University, Tel Aviv, Israel

Ofri Mosenzon

The Diabetes Unit, Department of Endocrinology and Metabolism,

Hadassah Ein Kerem Medical Center, P.O.B 12000,

9112001, Jerusalem, Israel

OFRIM@hadassah.org.il

Phone: +972-505172464

Fax +972-26424514

**Additional file 1 methods:**

Inclusion in the diabetes registry required at least one of the following five criteria:

1. **Diagnoses:** Active diagnosis or a visit diagnosis made by a primary care physician, a diabetes expert, an endocrinologist, or an ophthalmologist. This should be accompanied by at least one of the following
   1. Measurement of HbA1c≥6.5% within six months before or after receiving the diagnosis
   2. Two fasting plasma glucose tests of ≥126 mg/dL. One within six months before or after receiving the diagnosis, and the other without time constraints.
2. **Insulin:** Two insulin purchases on two different dates within three months. The registry does not capture females aged 18-55 years, without any of the other criteria, with a GTT or GCT test, with insulin purchases within a period shorter than four months.
3. **Oral glucose-lowering agents:** Two purchases on two different dates within three months, as well as an HbA1c measurement ≥6.5% and/or a fasting plasma glucose ≥126 mg/dL.
4. **HbA1c measurement:** Measurement of ≥7.25%.
5. **Plasma glucose measurement:** at least two measurements of ≥200mg/dL, at least 30 days apart**.**

**Figure S1: CONSORT diagram of subjects included in this analysis**

MHS, Maccabi healthcare services


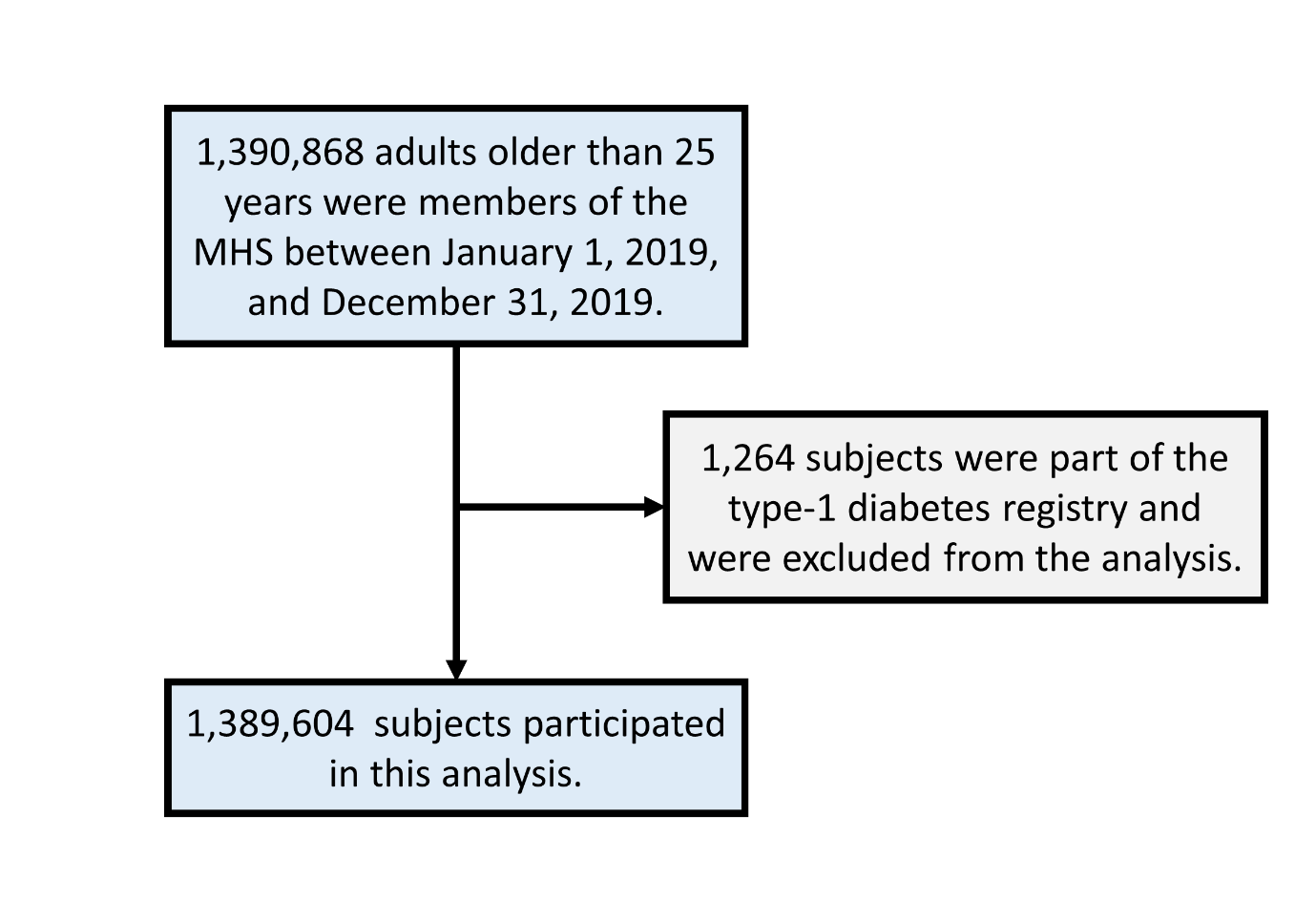


**Figure S2: Distribution by age of T2D, heart failure, and eGFR<60 mL/min/1.73 m^2^.**

Numbers indicate the percentage of patients within the specified age group, out of the total number of patients with heart failure, T2D or eGFR<60 mL/min/1.73 m^2^ in the MHS, as specified.

T2D, Type-2 diabetes; HF, heart failure; eGFR, estimated glomerular filtration rate.


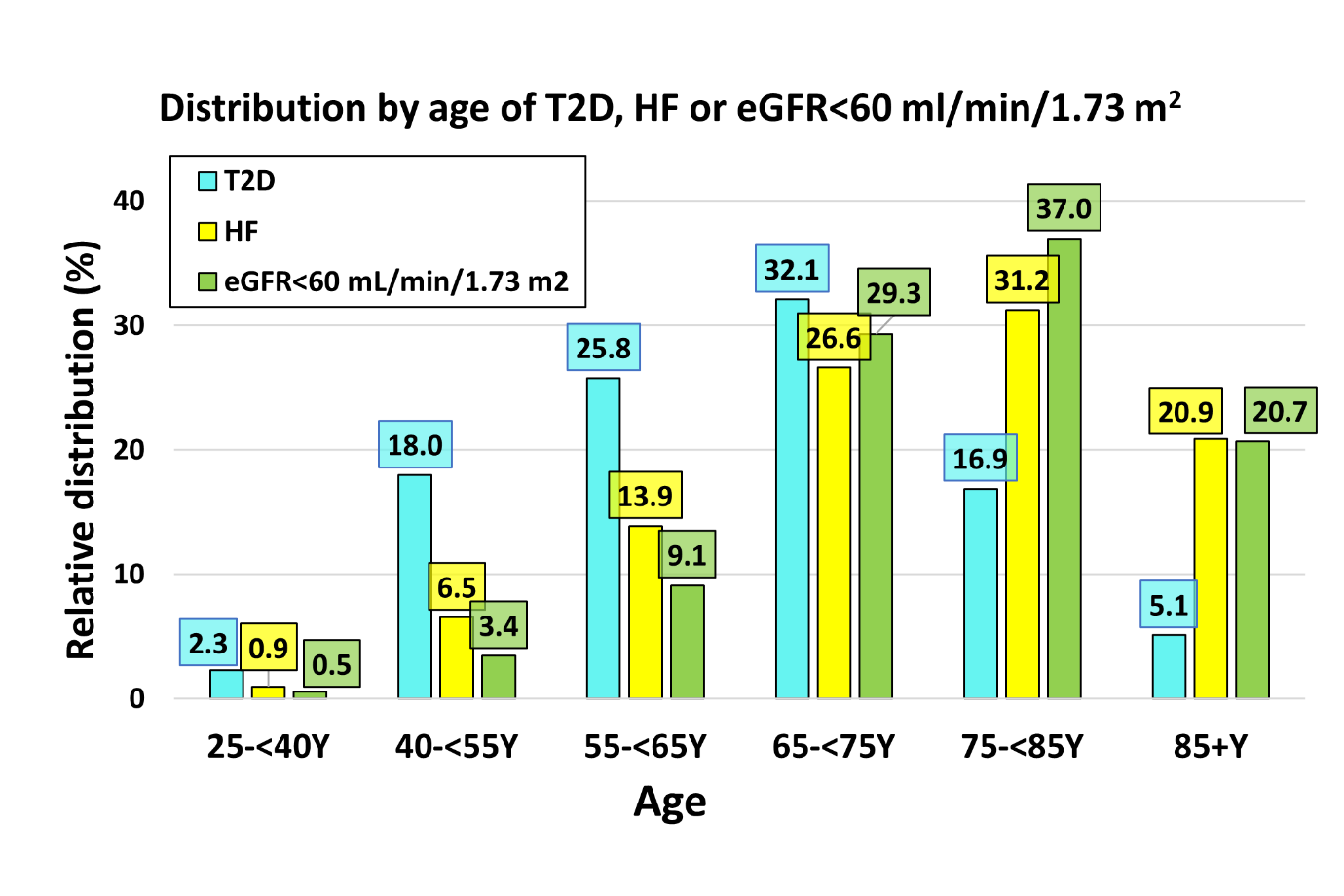


**Figure S3: Prevalence of T2D in patients with heart failure or eGFR<60 mL/min/1.73 m^2^ overall and at different age groups.**

Distribution of T2D in patients with heart failure (left) or with eGFR<60 mL/min/1.73 m^2^ (right) overall and at different age groups. In horizontal grey line – prevalence of patients *without* T2D in the overall study population. Numbers indicate the percentage of patients *without* a T2D diagnosis.

T2D, Type-2 diabetes; eGFR, estimated glomerular filtration rate.


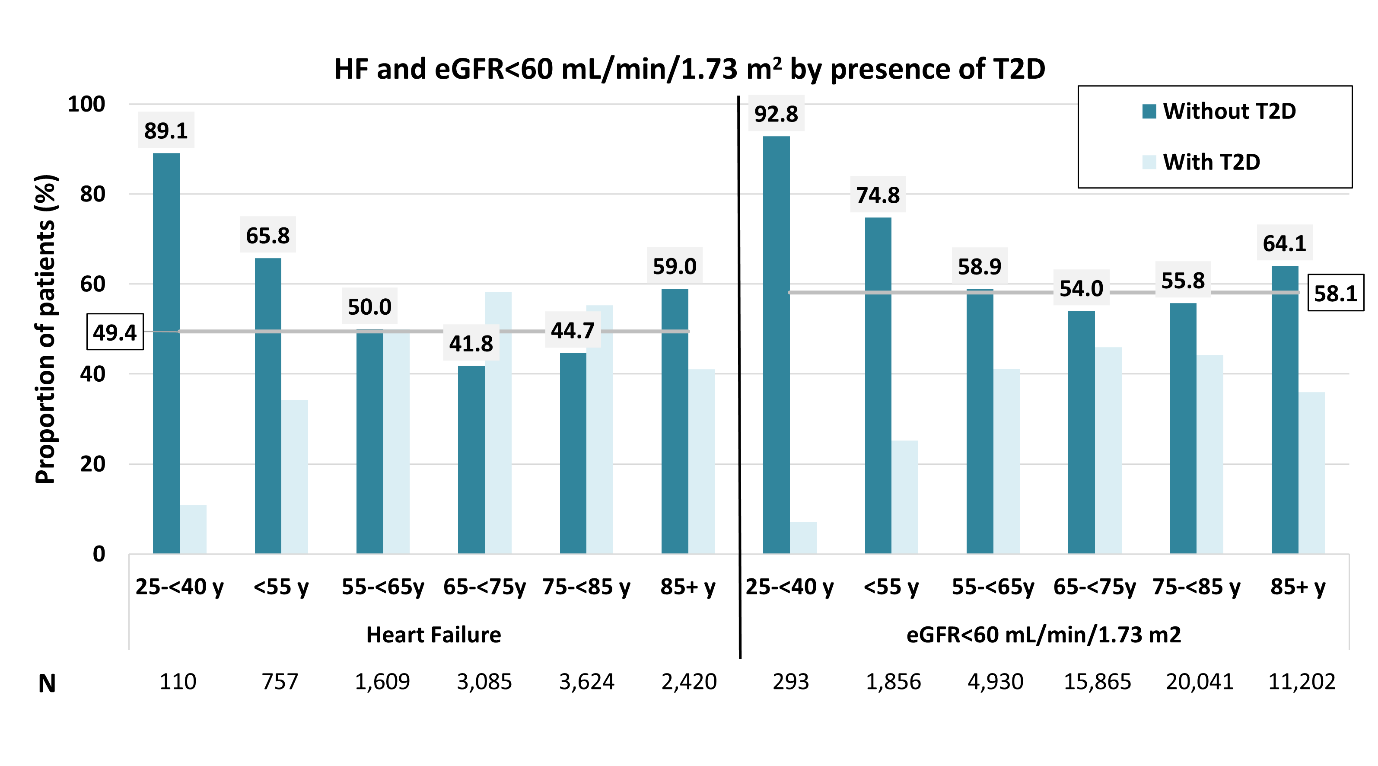


**Figure S4: Prevalence of heart failure by age, presence of T2D and kidney functions – in female and male participants separately.**

Numbers indicate the percentage of patients out of the entire sex-/age-/T2D-/eGFR- matched population in the MHS. Greener indicates lower prevalence, redder indicates high prevalence. White (reference) represents the overall prevalence in the study population (0.84%).

eGFR categories are in mL/min/1.73 m^2^.

T2D, Type-2 diabetes; HF, heart failure; eGFR, estimated glomerular filtration rate; MHS, Maccabi Healthcare Services


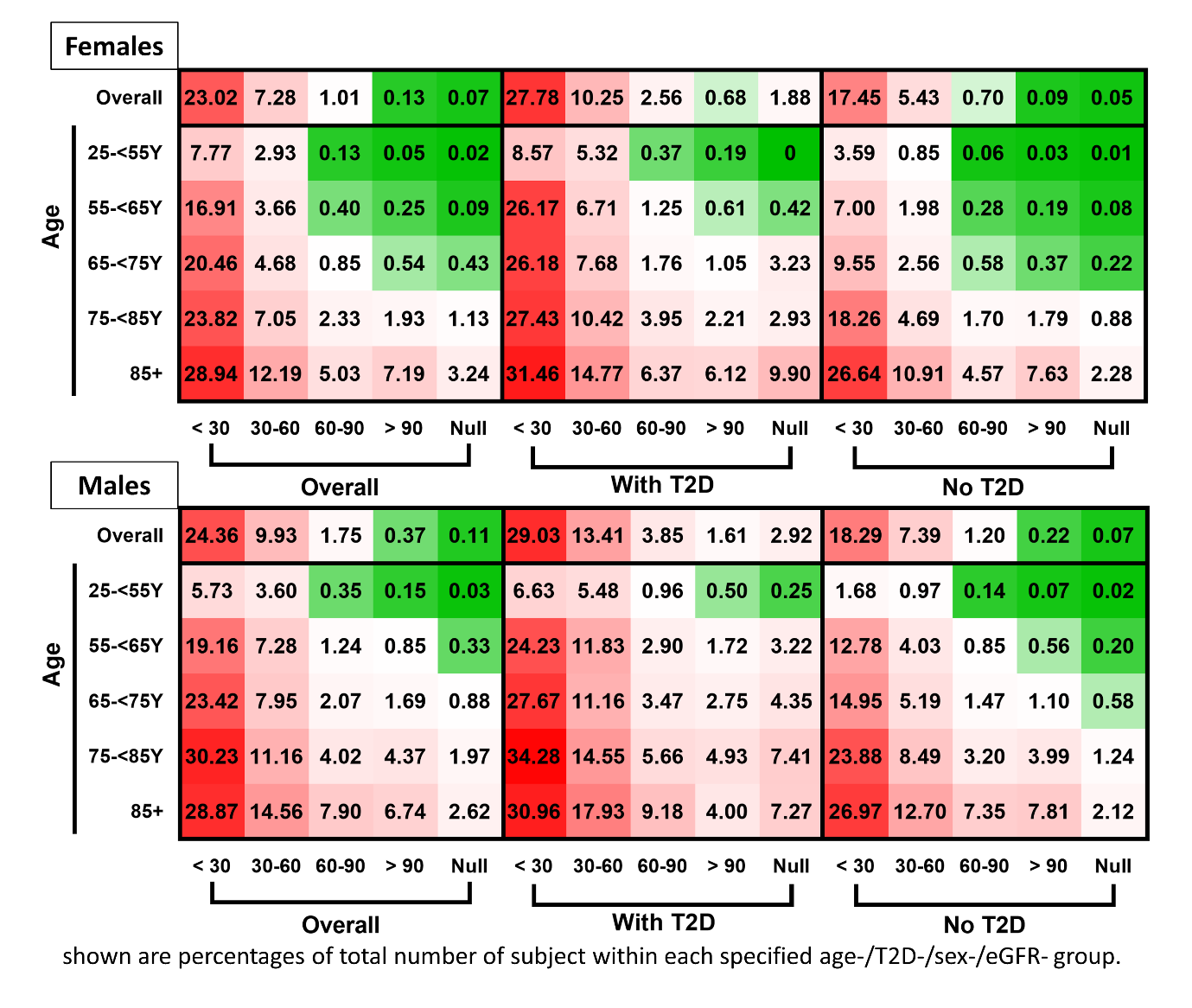


**Figure S5: Distribution of patients within different kidney functions categories by presence of T2D, heart failure, or neither.**

**A.** Distribution of eGFR values in the MHS population by presence of heart failure, T2D, or neither, presented as percentage of the entire cohort (all) and of those with an available eGFR value in the past 2 years (avlable).

**B.** Distribution of eGFR values in the MHS population by presence of heart failure, T2D or neither, by age groups, presented as percentage of the entire cohort (all).

eGFR values are presented in mL/min/1.73 m^2^

T2D, Type-2 diabetes; HF, heart failure; eGFR, estimated glomerular filtration rate; MHS, Maccabi Healthcare Services.


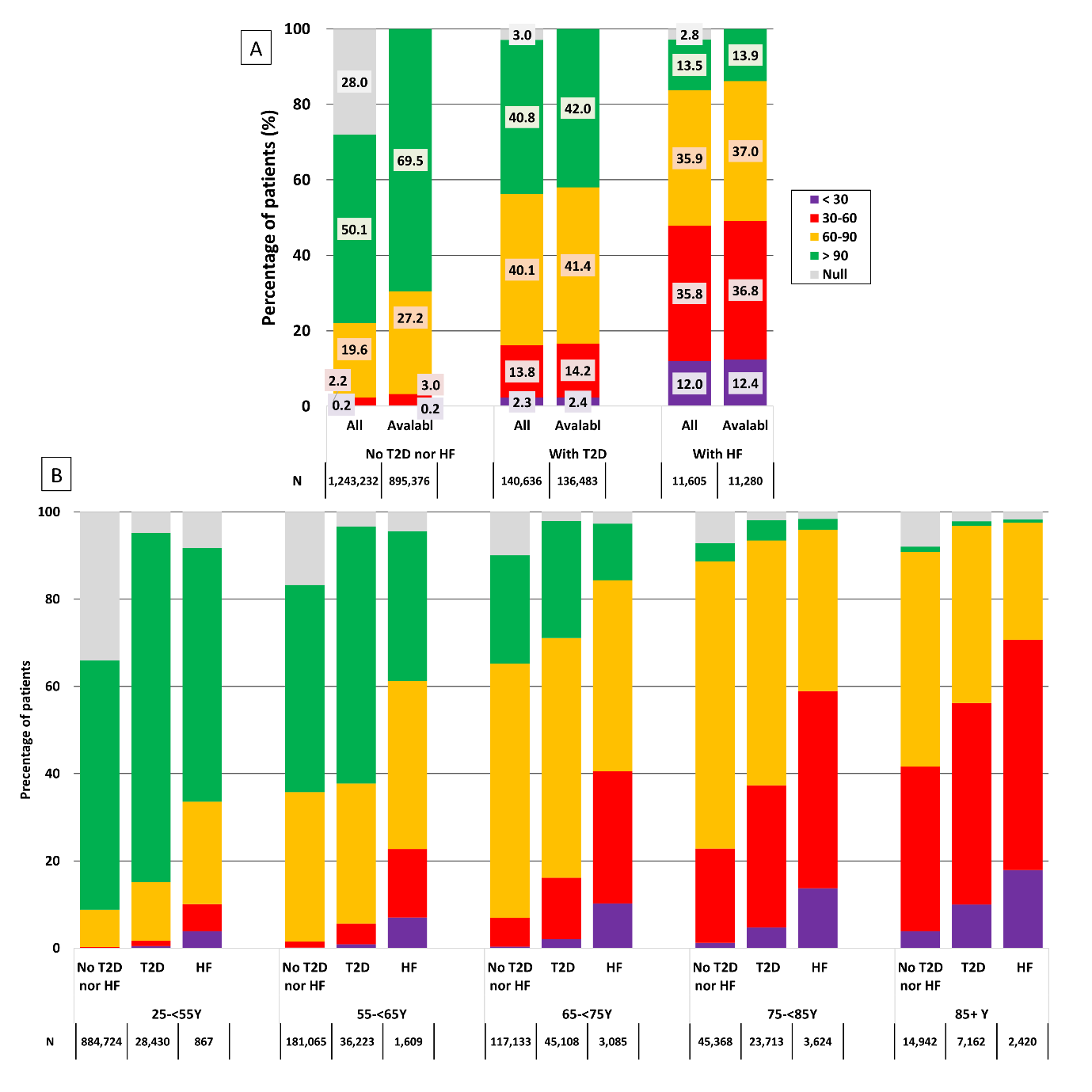


**Figure S6: Distribution of kidney functions in patients with heart failure by the presence of T2D.**

**A**. Distribution of eGFR values in patients with heart failure by the presence of T2D, overall, and by sex.

**B.** Distribution of eGFR values in patients with heart failure by the presence of T2D across age subgroups.

eGFR values are presented in mL/min/1.73 m2

T2D, Type-2 diabetes; HF, heart failure; eGFR, estimated glomerular filtration rate


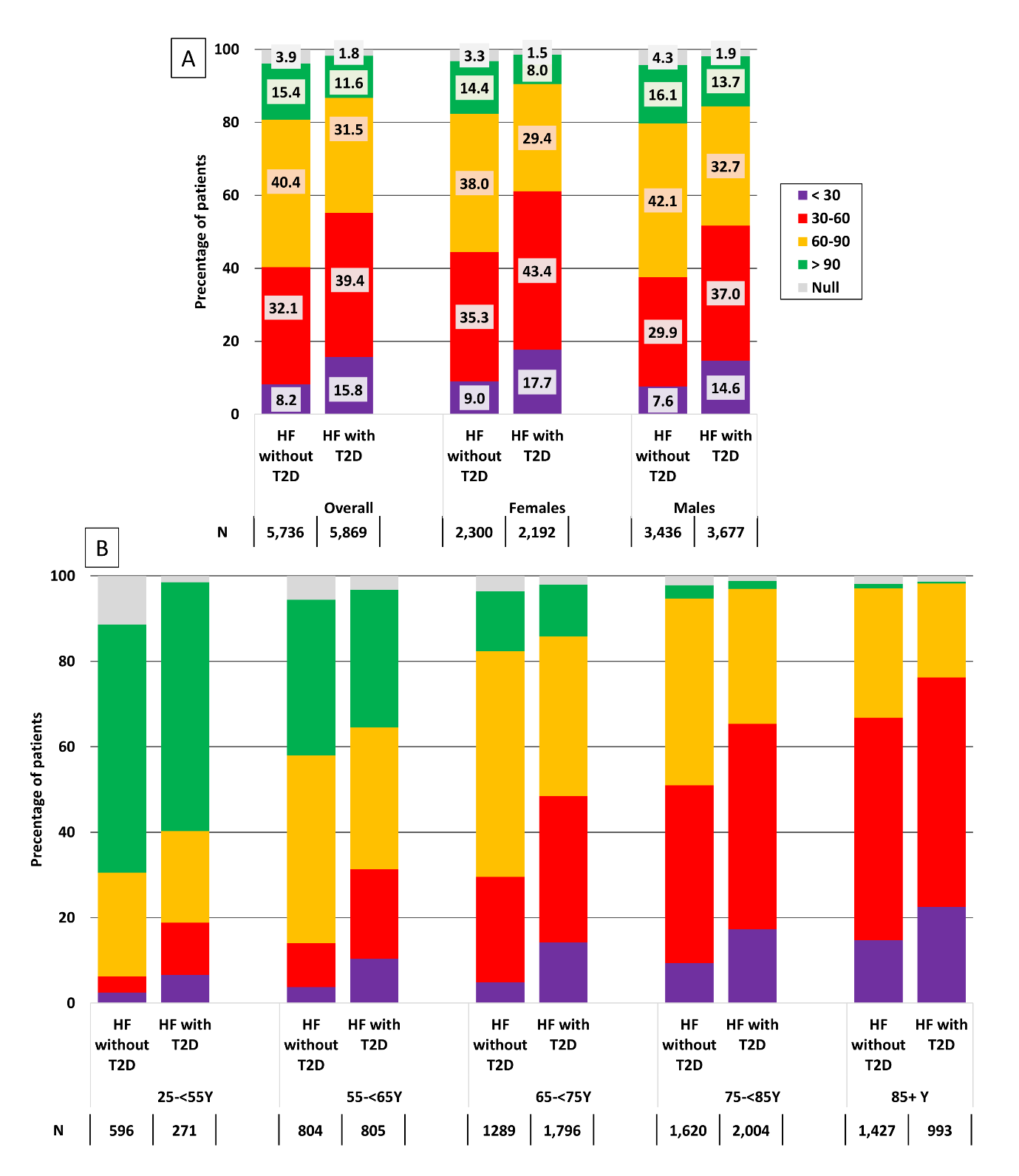

Supplement: Supplementary file 1 — Additional file 1: Figure S1. CONSORT diagram of subjects included in this analysis. Figure S2. Distribution by age of T2D, heart failure, and eGFR<60 mL/min/1.73 m2. Figure S3. Prevalence of T2D in patients with heart failure or eGFR<60 mL/min/1.73 m2 overall and at different age groups. Figure S4. Prevalence of heart failure by age, presence of T2D and kidney functions—in female and male participants separately. Figure S5. Distribution of patients within different kidney functions categories by presence of T2D, heart failure, or neither. Figure S6. Distribution of kidney functions in patients with heart failure by the presence of T2D. [file 12933_2022_1521_MOESM1_ESM.docx]
